# Transforming Prosthodontics and oral implantology using robotics and artificial intelligence

**DOI:** 10.3389/froh.2024.1442100

**Published:** 2024-07-29

**Authors:** Atharva P. Karnik, Harsita Chhajer, Swapna B. Venkatesh

**Affiliations:** Department of Prosthodontics and Crown & Bridge, Manipal College of Dental Sciences, Manipal Academy of Higher Education (MAHE), Manipal, India

**Keywords:** artificial intelligence, AI, machine learning, deep learning, robotics

## Abstract

The current review focuses on how artificial intelligence (AI) and robotics can be applied to the field of Prosthodontics and oral implantology. The classification and methodologies of AI and application of AI and robotics in various aspects of Prosthodontics is summarized. The role of AI has potentially expanded in dentistry. It plays a vital role in data management, diagnosis, and treatment planning and administrative tasks. It has widespread applications in Prosthodontics owing to its immense diagnostic capability and possible therapeutic application. AI and robotics are next-generation technologies that are opening new avenues of growth and exploration for Prosthodontics. The current surge in digital human-centered automation has greatly benefited the dental field, as it transforms towards a new robotic, machine learning, and artificial intelligence era. The application of robotics and AI in the dental field aims to improve dependability, accuracy, precision, and efficiency by enabling the widespread adoption of cutting-edge dental technologies in future. Hence, the objective of the current review was to represent literature relevant to the applications of robotics and AI and in the context of diagnosis and clinical decision-making and predict successful treatment in Prosthodontics and oral implantology.

## 1 Introduction

There is a rapid change happening in the field of Prosthodontics with newer inventions, concepts and technologies and materials. Incorporation of these changes into practice, education and research has allowed Prosthodontics to evolve in response to changing needs. Among the recent developments, there has been the introduction of artificial intelligence (AI) and robots in Prosthodontics. The idea of robotics was first applied in 1969. Automation of any tasks in laborious jobs can be done due to technological advancements in robotics and artificial intelligence. Robotics has therefore entered the operating room, and its uses are evolving continuously (1, 2). Training robots were introduced in dentistry to help the students to get experience of human-like machines which proved much better than the earlier used mummies. The first artificially intelligent robot created was called Showa Hanako and could mimic human gestures and reactions.

Artificial intelligence is the branch of Computer Science that creates smart machines that can carry out human tasks. It is the ability of a computer or computer-controlled robot to perform human activities. AI has allowed robots to mimic human cognitive functions such as perceiving, navigating, learning from experience (past data), and making decisions. These robots can even make decisions in ambiguous situations that are too complex for humans to handle. Robots learn all these human processes using Machine Learning (ML) which is again a part of artificial intelligence. AI has given robots a computer vision to perform all these human tasks more efficiently compared to humans (3, 4).

Digital dental techniques are standardized constantly and are a part of regular treatment plans. Specifically, the use of CAD/CAM (computer-aided design and computer-aided manufacturing) has become a routine practice in clinical and laboratory settings. Dental digitization is still evolving, and new developments such as the use of artificial intelligence are starting to emerge (5, 6). In the last few decades, AI was in stages of inception, but at present AI is used as an adjuvant aid in many fields including healthcare and dentistry. This article explains in detail how robotics using AI is changing the face of Prosthodontics and Implantology.

### 1.1 Classification of AI and methodologies

**Figure 1:**
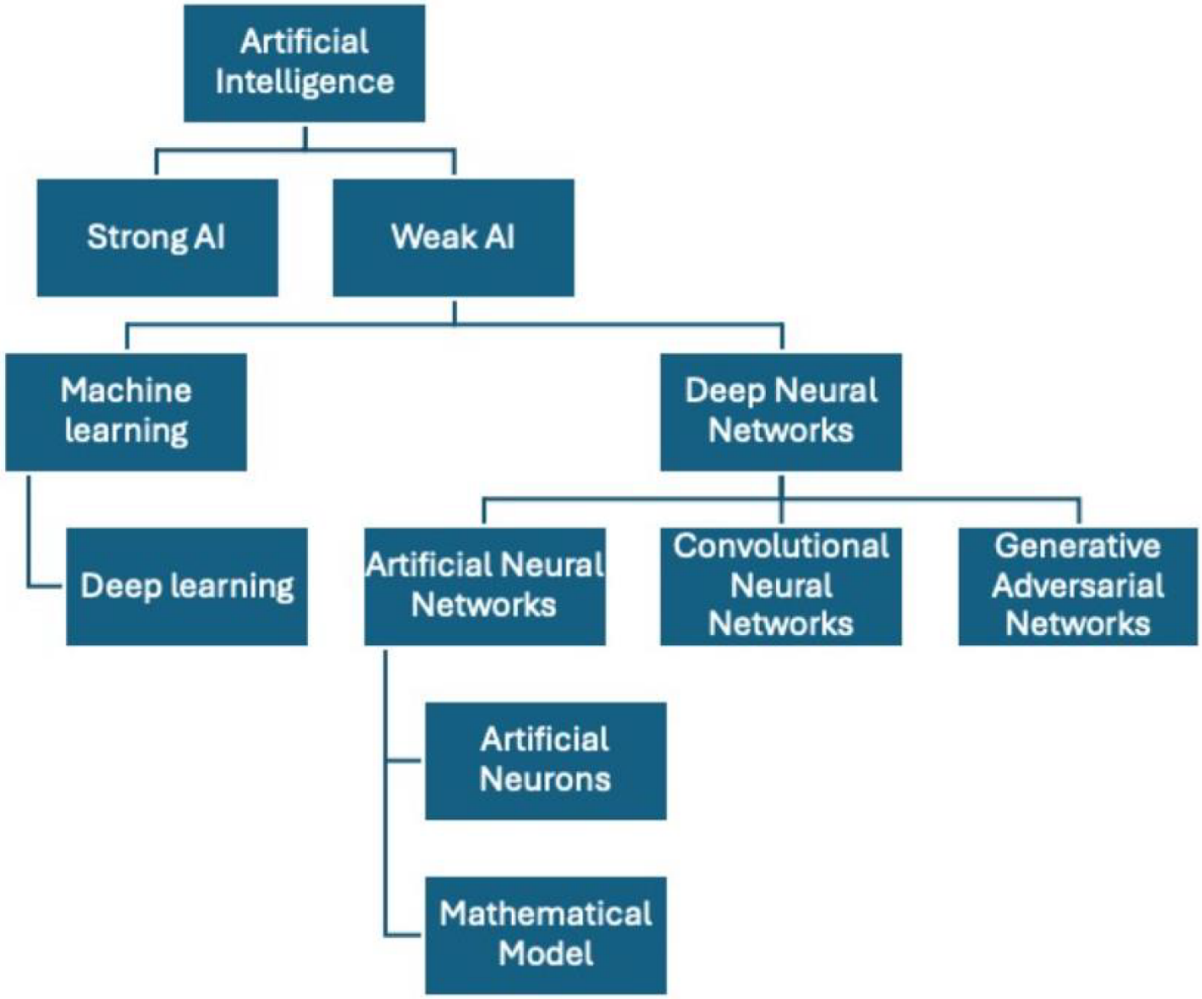
Schematic representation of types of artificial intelligence.

There are two types of AI: strong AI and weak AI. Strong AI refers to AI with human-like intellect and capabilities; it performs with the same flexibility and reactivity as people. The goal of strong AI is to create a multitasking algorithm that can make decisions in multiple domains. Weak AI relies on applying a program designed to accomplish a single or narrow set of tasks (7, 8) (Figure 1).

ML is a subclass of weak AI. Machine learning methods enable computers to identify patterns. The machine learning system is capable of making precise predictions on fresh data after it has been trained on preexisting datasets. In order for the algorithm to identify the required pattern, the training datasets frequently need to be made simpler. The degree to which these interventions are executed with precision determines how well ML systems operate (6, 9).

A popular type of machine learning at the moment is deep learning (DL). Deep learning refers to a “set of computational models composed of multiple layers of data processing, which make it possible to learn by representing these data through several levels of abstraction” (6, 9). Deep neural networks (DNNs) require more data than machine learning since they can individually learn from and organize the training data set in a hierarchical fashion. The three main types of deep learning systems are artificial neural networks (ANN), convolutional neural networks (CNN), and generative adversarial networks (GAN). The primary applications for all three models are in image production and recognition (6, 9).

Artificial neural networks mimic the networks of neurons seen in the human brain to understand and make judgments in a manner comparable to that of a person. ANN's primary components are artificial neurons, a mathematical model derived from human neuronal networks. Together, these layers of artificial neurons form a network that is integrated to do particular tasks, such as image categorization (e.g., radiographic image of decaying teeth) ANN gives an advantage of learning (10).

Lotfi Zadeh created fuzzy logic (FL) in 1965. Humans struggle to find solutions to the many nebulous challenges they encounter in their daily lives. In these kinds of circumstances, fuzzy logic is helpful for solving fuzzy problems. By enabling the use of more sophisticated decision-tree processing and improved integration with rules-based programming, the FL serves as the foundation for artificial intelligence. FL mimics how humans make decisions, which entails considering options in between the digital values YES and NO. It generates workable answers to vague and insufficient challenges (3, 4, 10).

### 1.2 Data mining in robotics

Data is the core of robotics. Data is a storehouse of knowledge and insights which can be processed using ML to extract relevant information. It can be utilized in the medical field to diagnose patients’ conditions. Robots handle a lot of data to carry out specified activities i.e., from data collection to the production of significant results (internal data). Data is accessible in a variety of formats. Thus, handling and organizing all this data is crucial. Data mining refers to the entire process of gathering data, cleaning it, and using it to produce insightful analyses and forecasts (4).

## 2 Applications of AI and robotics in Prosthodontics and dental implantology

Prosthodontics mainly focuses on the treatment of completely and partially edentulous conditions with fabrication of removable and fixed dental prosthesis or implants and construction of a maxillofacial prosthesis.

### 2.1 AI assisted diagnosis

AI application for accurate diagnosis in Prosthodontics is based on AI-based imaging analysis. Intra oral scanners and CBCT scans generate large amounts of digital data, AI algorithms can extract valuable information and assist in diagnosis. ML makes these functions possible by teaching computers rules based on data and identifying inherent statistical patterns and structures in the data and thus help in analyzing any anomalies in the tooth structure (11–13).

### 2.2 AI assisted treatment planning

AI-assisted treatment planning algorithms play a vital role in simplifying and optimizing the treatment planning process. These algorithms create personalized treatment regimens by analyzing patient data, such as clinical records, diagnostic pictures, and patient-specific characteristics, using artificial intelligence approaches. AI algorithms can identify patterns and correlations in a big dataset to establish the best course of action for each patient by utilizing machine learning and data mining. To create individualized treatment plans and optimize treatment outcomes, factors such as patient's current state of oral health, aesthetic preferences, functional requirements, and anatomical concerns are considered (11).

### 2.3 AI supported advances in CAD/CAM

AI enabled CAD software has revolutionized prosthodontic design by incorporating AI algorithms to improve the precision, effectiveness, and personalization of dental prosthesis design. AI algorithms can be used to evaluate intraoral scans, digital impressions, and 3D models, to design precisely fitting dental prosthesis. The overall effectiveness and precision of prosthodontic design procedures are enhanced by CAD software with AI capabilities, which automate some design tasks and make recommendations for changes based on historical data and anatomical considerations. Optimization of designing process can be done using ML algorithms which consider various factors such as occlusion, aesthetics, and functional requirements (12, 14). AI and CAD/CAM have improved the quality of removable dentures while optimizing laboratory procedures. Reducing or eliminating the amount of manual laboratory procedures allows the dentist and dental technician to ensure the accuracy and repeatability of the prosthesis. This results in a reduction of the overall time required for rehabilitation. AI is used to assist in creating the greatest and most aesthetic prosthesis for patients while considering variables like ethnicity, facial proportions, anthropological calculations, and patient's demand. CAD/CAM technology, subtractive milling, and additive manufacturing methods, such as 3D printing is used for fabricating the prosthesis (15). Henriette Lerner et al. introduced an AI approach to lessen the errors when cementing CAD/CAM crowns. This artificial intelligence model was intended to support the production of fixed implant prostheses with monolithic zirconia crowns. The AI model helped to identify the abutment's subgingival margins (16).

### 2.4 AI and Removable Prosthodontics

AI can be applied to 3D printing technology to fabricate removable partial dentures (RPD). AI algorithms can also help establish customized approach to RPD design by analyzing patient data to produce a design that is specific to each patient's needs and preferences and anatomy. AI can be used to evaluate the fit and function of RPD (17). A study developed an AI model with the help of CNN for classification of dental arches to assist in the fabrication of dentures (18).

### 2.5 AI and Fixed Prosthodontics

The precision and effectiveness of tooth preparation are increased with the application of AI. AI algorithms can learn and analyze from a vast database of effective crown designs, providing insights into the best contour and extension of finish line in the prepared teeth. AI can automate the extraction of marginal lines with precision thereby assisting in the tooth margin preparation, which generally requires advanced technical skills. A study used convolutional neural network model called Sparse Octree to extract accurate margins (19). Studies have mentioned the use of AI algorithms for ceramic shade selection included fuzzy logic, back-propagation neural networks, CNN, ANN, support vector machine algorithms (20).

### 2.6 AI and Maxillofacial Prosthesis

The prosthetic devices powered by AI can assist patients with maxillofacial anomalies or injuries in restoring both esthetics and their function by mimicking human neurons through CNNs. AI powered artificial eyes, for example, can let patients see without surgery, while voice-activated smart reading glasses can help the visually impaired read text and recognize faces (21). AI has also been used in tissue engineering to create skin replacements for wound healing. Artificial skin grafts serve as long-term skin replacements or transient wound dressings. They are primarily used to supply oxygen, avoid dehydration, encourage healing, and guard against infections (22).

### 2.7 AI and Implant Prosthodontics

CNN models can be used to classify implants using periapical and panoramic radiographs. The bone quality and quantity can be assessed by using AI algorithms using CBCT images, the clinicians can measure the amount of bone loss more accurately and detect areas of potential bone loss (6, 23). A 3D model of the patient's jawbone can be created using AI from CBCT pictures, which helps in implant design and placement. This can assist in determining the ideal site and angle for placement of implant, thereby improving the success rate (24).

Digital planning software can be used by clinicians to generate a virtual surgical guide to assist with implant placement during surgery. Fabrication of physical surgical guide using rapid prototyping can be utilized during surgery to ensure precise implant placement (17, 25). AI in implant surgical planning helps in being precise and effective by enabling the creation of personalized treatment plans for individual patients by combining multiple scan and data. This can enhance patient outcomes and guarantee the success of the implant surgery (17, 26).

### 2.8 AI and digital smile designing

AI generated smile designing software can be used for smile designing. These software's are highly accurate, generating natural simulated images, facilitating engagement of the patient and case acceptance, enhances patient satisfaction, enhances connection and efficiency in teamwork. Using complex algorithms, the software analyzes the patient's facial features, such as facial symmetry, lip line, and tooth size and form. A virtual patient is created using several images, intraoral and CBCT scans entered in the program. The ability to simulate, customize, and test the results allows the patient and the dentist to both completely understand the situation before proceeding with planned dentition modifications (27, 28).

## 3 Application of robotics

### 3.1 Tooth arrangement robots for complete dentures

The most important step in the fabrication of conventional complete dentures is arrangement of teeth in centric occlusion, which is effectively done by a specialized dentist or a skilled dental technician. The conventional way of fabricating denture systems has been superseded by robotic fabrication. Complete dentures range greatly in terms of tooth size, dental arch curvature, and the placement and alignment of individual teeth relative to one another. The advantage with robots is the flexibility which can be configured to fabricate dentures (29). A single manipulator robotic system developed by CRS robots manufactured in Canada is used for the fabrication of complete denture prosthesis via 6 DOF (Degree of Freedom). The three-dimensional virtual tooth arrangement software of the robot uses patients mandibular arch parameters to create patients’ medical history data, expert-drawn jaw, and dental arch curves. Following the three-dimensional display of the proposed dentitions, an interactive virtual observation environment is offered, allowing users to modify each tooth's position. This system is based on the use of a unique photosensitive substance that hardens when illuminated, which selected standard teeth and fixed in a standard position. Nevertheless, it was discovered that artificial teeth were difficult for the system to precisely grip and manipulate. Advanced robotic systems with more DOF were developed (30, 31).

### 3.2 Tooth preparation robot

Yuan et al. presented a robotic system for tooth preparation that included the following hardware components: (a) a dental fixture that joins the robotic tool to the target tooth and shields the nearby teeth from the laser's cutting.; (b) a 6 degree of freedom robotic arm; (c) an efficient low-heat laser for hard tissue preparation; (d) CAD/CAM software to create the target form for tooth preparation and to produce a 3D motion path for the laser (e) an intraoral 3D scanning device to acquire 3D data about the target tooth, neighboring teeth, and the subject's dental fixture (32). The outcomes showed improved performance when compared to the clinician's crown preparation using the robotic system's average repetition ability (33).

### 3.3 Robots in Dental Implantology

Robotic implant systems typically need real time surgical tracking for accurate implant placement. The First robot-guided dental implant placement was presented by Boesecke et al. in 2002. The working region scope of robot system was 70 cm, could execute drilling guide for implant osteotomy, and could place 48 dental implants within 1–2 mm of the apical border. In addition, a 3-DOF robotic system with a stereo camera was developed. This system could recognize and control the dental handpiece, ensuring that implants were placed in accordance with the preoperative protocol. The computer automatically applied the planned surgical process to guarantee the right cutting site and applied the right amount of force (1, 2). The first computerized navigation robotic system with FDA approval to improve the clinical accuracy of dental implant surgery is called YOMITM (*N*eocis, Miami, FL, USA), introduced in 2017. It is a computerized navigation device designed to assist dental implant surgery in both the pre-operative and intra-operative phases. Vibrational feedback is used by the navigation system to prepare dental implant osteotomy with excellent predictability and precision. By offering physical guidance for the depth of the drill, position and orientation, YOMI helped to prevent the need for a customized surgical guide and deviation of operator's hand. Zhao unveiled the first automated implant placing technology in history in 2017. Surgical procedures can be altered automatically and with a high degree of autonomy, and treatments can be carried out without the need for a dentist's intervention. Nevertheless, there aren't many validation data available for the robot's intelligent decision-making or the viability and dependability of implant placing. Autonomous dental implant robot was also developed in 2017 by Fourth Military Medical University Hospital (Xi'an, China) and Beijing University which prevented errors during surgery and addressed the shortage of competent dentists. Robotically manufactured surgical guides are less costly, less intrusive, and less likely to result in human error during clinical procedures. They also provide complete control over the trajectory of the implant (1, 2, 34).

An innovative development in oral healthcare is the integration of robotics and AI into implant dentistry. Robots can optimize implants placements as they are equipped with precise algorithms, thereby enhancing longevity and effectiveness of the implant prosthesis. AI complements this by analyzing large datasets, facilitating evidence-based, customized treatment plans, and offering crucial support for anything from surgical feedback to diagnostics. AI algorithms can identify patterns, foresee potential complications, and suggest the most accurate implant designs, optimizing the overall treatment process. Robotic systems have been used in performing complex implant surgeries with high precision, thereby increasing patient satisfaction and implant survival rates. AI algorithms have made it easier to accurately identify the quantity and quality of bone, which has improved implant placement and decreased the chance of implant failure. These developments have made it possible to reduce surgical time, achieve better esthetic results, and provide greater patient comfort (1).

### 3.4 Robots in Gnathology

Waseda Yamanashi developed a master-slave system to facilitate training of mouth opening. Jaw movements can be replicated by robotic articulators with or without the assistance of jaw movement tracking sensors. A full veneer crown was created in an experiment on the working cast of a single patient utilizing a robotic articulator. The most recent development is a commercially available robot, called the “Bionic Jaw Motion” (Bionic Technology, Vercelli, Italy). The robot uses a combination of a robot articulator and a movement analyzer (a high-speed camera) to replicate mandibular kinematics (35).

### 3.5 Robots for treating TMJ disorders

The Waseda Asahi Oral-rehabilitation robot No. 1 (WAO1) is an oral rehabilitation robot developed by Waseda University in Japan. Comprising a headrest-equipped body, two arms with six degrees of freedom and plungers, a control box, a computer, and an automated massage trajectory generation system with virtual compliance control, the robotic apparatus massages the patient's face by pressing or rubbing a plunger whose action is automatically controlled by a computer. The first robot to massage a patient's facial tissues, masticatory muscles (masseter and temporalis), and oral structures like the parotid gland and duct, is the WAO1. It can therefore be used to treat dry mouth and TMJ disorders (36).

### 3.6 Speech robots

This was developed in 2005 at the university in the Canadian province. There are two 3-DOF parallel manipulators that drive the two TMJs, one at each end of the jaw. The purpose is to examine how jaw movements impact our ability to receive and understand facial communication (36).

## 4 Limitations of AI and robotics

Integration of AI in Prosthodontics causes concerns about patient privacy and confidentiality, as well as the ethical implications of AI-generated diagnoses or treatment plans. AI may have biases built into its programming that provide unfair or discriminating results. Concerns are also raised about the potential loss of jobs for dental technicians, as AI takes over their tasks (37). While using AI to solve issues, a thorough algorithm with multiple applications is required to answer a particular question. Artificial intelligence cannot provide direct interpretation; instead, errors in algorithms may lead to misunderstandings. If the diagnostic task grows overly reliant on the AI system, there will also be a growing risk of responsibility (38). Practitioners may have doubts regarding the workings of the AI software, they do not understand and may compromise its clinical significance. Clinicians should always exercise caution and vigilance when evaluating data provided by AI. Another crucial factor in dentistry is data misuse and AI security concerns. Entrusting a computer with decisions about human health and relying only on the machine to make decisions about health care services is crucial (37).

Robots have proven to be efficient, accurate, and repeatable. The introduction of this novel technology into existing clinical set up may have several obstacles of varying nature. One of the obstacles the dentist faces is that the applications are very expensive. The robotic systems are complex, specialized knowledge and expertise is needed to ensure optimal performance (1). Dentist may lack of knowledge and expertise to program and control the robotic systems. Furthermore, dentists’ acceptance and compliance with the unknown patient may be another crucial factor (4). The research in the field of robotics dentistry is limited due to the lack of accessible systems and lack of expertise to program and regulate robotic systems. The research in the field of robotics relies on effective collaboration between engineers and dentists (39).

## 5 Conclusion

AI and robotics are emerging fields in dentistry. Dentistry is progressing toward a new era of robotically assisted and data-driven care. Robotic devices and 3D monitoring will become routine in dental setup and continue to enhance patient care as technology is incorporated into clinical practice. Nevertheless, the most recent advancements in AI and robotics have not yet been fully integrated into research in dentistry, nor have they reached a point of technological readiness and economic viability where they can be commercialized.
